# Efficient quasi-monoenergetic ion beams from laser-driven relativistic plasmas

**DOI:** 10.1038/ncomms10170

**Published:** 2015-12-11

**Authors:** Sasi Palaniyappan, Chengkun Huang, Donald C. Gautier, Christopher E. Hamilton, Miguel A. Santiago, Christian Kreuzer, Adam B. Sefkow, Rahul C. Shah, Juan C. Fernández

**Affiliations:** 1 Los Alamos National Laboratory, Los Alamos, New Mexico 87545, USA; 2 Ludwig-Maximilian-University, Munich, Germany; 3 Sandia National Laboratory, Albuquerque, New Mexico 87185, USA

## Abstract

Table-top laser–plasma ion accelerators have many exciting applications, many of which require ion beams with simultaneous narrow energy spread and high conversion efficiency. However, achieving these requirements has been elusive. Here we report the experimental demonstration of laser-driven ion beams with narrow energy spread and energies up to 18 MeV per nucleon and ∼5% conversion efficiency (that is 4 J out of 80-J laser). Using computer simulations we identify a self-organizing scheme that reduces the ion energy spread after the laser exits the plasma through persisting self-generated plasma electric (∼10^12^ V m^−1^) and magnetic (∼10^4^ T) fields. These results contribute to the development of next generation compact accelerators suitable for many applications such as isochoric heating for ion-fast ignition and producing warm dense matter for basic science.

## Abstract (web summary)

Table-top laser-plasma ion accelerators have many potential applications, but achieving simultaneous narrow energy spread and high efficiency remains a challenge. Here, the authors produce ion beams with up to 18 MeV per nucleon whilst keeping the energy spread reduced through a self-organized process.

## Graphical abstract

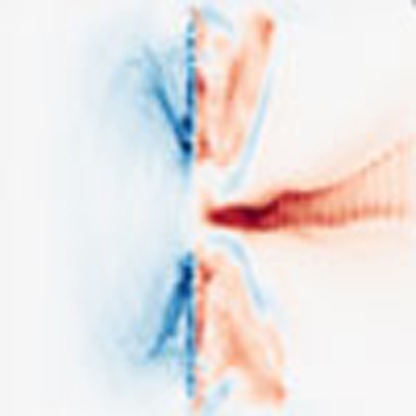

Laser-driven ion beams with narrow energy spread and high conversion efficiency would be transformational because they could deliver unprecedented power densities. For example, warm dense matter with conditions relevant to stars and planetary cores can be created in the laboratory by isochoric heating of bulk matter with such ion beams^1^,^2^. Ion-fast ignition is an extreme example of isochoric heating, where laser-driven ion beams can ignite compressed fuel to generate fusion energy^3^,^4^,^5^,^6^,^7^.

Despite a decade-plus effort, achieving laser-driven ion beams with simultaneous narrow energy spread and high efficiency remains a big challenge^8^,^9^,^10^,^11^,^12^,^13^,^14^,^15^,^16^,^17^,^18^,^19^,^20^. Widely explored schemes for laser-driven ion beam generation include target normal sheath acceleration (TNSA)^9^,^10^,^11^,^21^,^22^,^23^,^24^,^25^,^26^,^27^,^28^,^29^, radiation pressure acceleration (RPA)^12^,^13^,^14^,^20^,^30^,^31^,^32^,^33^, coherent acceleration of ions by laser (CAIL)^34^,^35^, breakout afterburner (BOA)^36^,^37^,^38^, magnetic vortex acceleration (MVA)^39^,^40^,^41^,^42^ and collisionless shock acceleration (CSA)^16^,^17^. Broadly, these attempts can be classified into three categories, viz., (1) picosecond laser pulses interacting with microns-thick opaque targets, mostly TNSA, (2) ultrashort femtosecond laser pulses interacting with either ultrathin solid targets such as RPA and CAIL or near-critical/gas targets such as MVA and (3) the intermediate regime of picosecond laser pulses interacting with either thin sub-micron solid targets in the relativistic transparency (RT) regime such as BOA, or gas targets such as CSA.

The TNSA mechanism typically produces a broad exponential ion energy spectrum^21^,^22^,^23^,^24^,^25^,^26^,^27^,^29^. In 2006, Schwoerer *et al.*^10^ reported 1.2 MeV quasi-monoenergetic proton beam with ∼40% energy spread and Hegelich *et al.*^11^ reported 3 MeV per nucleon quasi-monoenergetic carbon ion beam with 17% energy spread, both under TNSA scheme using complex engineered targets with thin coating on the rear side (category 1). Nevertheless their conversion efficiency was much <1% (refs 9, 10, 11). In 2009, Henig *et al.*^13^ reported quasi-monoenergetic 2.5 MeV per nucleon carbon ion beam with ∼35% energy spread and 2.5% conversion efficiency (carbon ion beam contained 35 mJ of energy) when a 30-TW Ti:Sapphire ultrashort laser pulse interacted with a 5-nm-thick diamond-like carbon foil (category 2). In 2011, Haberberger *et al.*^16^ reported quasi-monoenergetic 20 MeV protons with 1% energy spread and efficiency much <1% when multi-TW Neptune CO_2_ laser pulses interacted with a hydrogen gas jet (category 3). In 2012, Kar *et al.*^14^ reported quasi-monoenergetic ∼7 MeV per nucleon carbon ion beam with ∼60% energy spread and 1% conversion efficiency when a 0.25 PW Vulcan picosecond laser pulse (∼200 J, ∼800 fs) interacted with a 50-nm-thick copper foil (category 3). These results illustrate the tremendous progress in generating efficient quasi-monoenergetic ion beams from laser-driven plasmas. However, applications such as ion-fast ignition require quasi-monoenergetic ion beams at tens of MeV/nucleon with efficiency ∼10%.

Here we report ion beams with narrow spectral peaks at energies up to 310 MeV for Al^11+^ (11.5 MeV per nucleon) and 220 MeV for C^6+^ (18.3 MeV per nucleon) with ∼5% conversion efficiency from relativistic laser–plasma interaction in category 3. Also, we demonstrate that by increasing the focused laser intensity fourfold (by reducing the focusing optic *f*-number twofold), the spectral-peak energy increases twofold. These results are obtained with the 0.12 PW (80 J, 650 fs, linear polarized) Trident laser^43^ at the Los Alamos National Laboratory, irradiating planar foils of an optimized thickness of up to 250 nm. Although the experimental set-up here is similar to prior ones at Trident laser facility, the new results demonstrated here are primarily due to reduced target pre-expansion and optimization of the onset of relativistic transparency in dense target plasmas that enables (according to simulation) a self-organized process which reduces the ion beam energy spread (discussed later).

## Results

### Overview

Table 1 summarizes the laser/target parameters along with the resulting plasma and ion beam properties for seven different sets of experiments (rows 2–8) discussed in this article. Experiments I, II, IV, V and VII indicate the conditions under which ion spectral peak is generated.

### Quasi-monoenergetic ions from relativistically transparent plasmas

**Figure 1:**
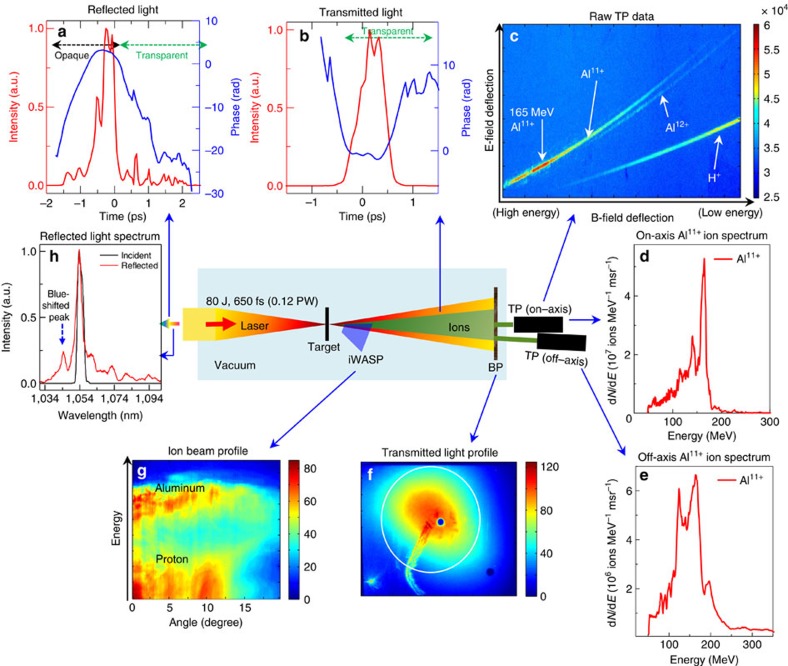
Schematic of experimental set-up (*f*/3 laser focus onto 110 nm Al foil). The 0.12 PW Trident laser is focused with *f*/3 off-axis parabola (peak intensity—2 × 10^20^ W cm^−2^) onto a 110-nm-thick aluminium foil (Table 1—Experiment I). (**a**) Time-resolved reflected light intensity (solid red line) and its temporal phase (solid blue line) measured using a single-shot frequency-resolved-optical-gating (FROG) showing the onset of relativistic transparency. The positive (negative) slope of the temporal phases indicate spectral blue (red) shift via *ω*_*t*_=d*φ*/d*t*, where *ω*_*t*_ is the instantaneous angular frequency; (**b**) time-resolved transmitted light intensity (solid red line) and its temporal phase (solid blue line) measured using a separate FROG showing the phase reversal during relativistic transparency; (**c**) raw Thomson parabola (TP) data; (**d**) measured Al^11+^ ion spectrum on-axis (solid red line) with 166 MeV spectral peak; (**e**) measured Al^11+^ ion spectrum 8.5° off-axis (solid red line) with 165 MeV spectral peak. TP used image plate (IP) detectors cross-calibrated against CR-39 nuclear track detectors. The TP instrumental cut-off is at 50 MeV due to an 18-μm-thick aluminium foil placed in front of the ion detector to block laser light; (**f**) transmitted laser beam profile captured on a Macor plate (30.5 × 30.5 cm square) and imaged onto a separate CCD camera (Apogee Alta U8300). This is used to quantify the amount of laser energy transmitted through the plasma; (**g**) angularly resolved ion energy spectra measured using an ion wide-angle magnetic spectrometer (iWASP); (**h**) reflected light spectrum (solid red line) measured using an infra-red spectrometer (Bruker Optics with Andor iDUS 1.7 μm InGaAs CCD) along with incident laser spectrum (solid black line) show a blue-shifted spectral peak.

Figure 1 shows schematic layout of the experiment. An *f*/3 off-axis parabola focuses the 80 J, 0.12 PW Trident laser (peak laser intensity of 2 × 10^20^ W cm^−2^) onto the target, such as a 110-nm-thick aluminium foil (density—2.7 g cm^−3^) (Table 1—experiment I). Multiple optical and particle diagnostics were used to characterize the experiment. The sharp drop in reflected laser-light intensity (Fig. 1a) near the peak of the main laser pulse indicates the ensuing relativistic transparency phase in the laser–plasma interaction. The temporal phase of the transmitted laser light (Fig. 1b) indicates the evolution of the electron density and relativistic *γ* factor during relativistic transparency phase consistent with earlier results^44^. Since the density is monotonically decreasing, the temporal phase reversal reflects the peaking of *γ*, in agreement with simulation. In these shots, the plasma turned relativistically transparent near the peak of the laser pulse. Empirically this appears to be a necessary condition to realize accelerated ion spectral peaks with the Trident laser pulse. The transmitted laser beam (Fig. 1f) contains 20% of the incident laser energy (that is 16 J). Relativistic transparency enables strong volumetric laser coupling into the dense plasma.

The time-integrated reflected light spectrum (Fig. 1h) from the aluminium foil shows a 9-nm blue-shifted peak at 1,044 nm. This Doppler shift d*λ*/*λ*=2*v*/*c*≅−0.009 indicates that the plasma critical surface expands at a speed of 1.4 μm ps^−1^ towards the incoming laser. The Doppler shift in the temporal phase of the backscattered FROG measurement provides the instantaneous velocity of the plasma critical surface (*v*_*cs*_) at the front side of the plasma 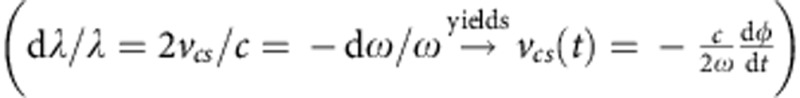. The time-resolved temporal phase of the reflected light from aluminium (Fig. 1a) shows an early positive phase slope (that is frequency blue-shift) d*ω*/*ω*=0.011 from *t*=−1.75 ps to *t*=−0.75 ps. This corresponds to a plasma critical surface expansion of 1.7 μm ps^−1^ towards the laser. It is important to remark that the backscattered light from the laser-pulse pedestal at the nanosecond timescale is too dim, and therefore these diagnostics are only sensitive to backscattered light during the rising portion of the main pulse. The plasma expansion velocity during the main laser pulse is inversely correlated to the target pre-expansion during the nanosecond pedestal. This is discussed in detail later under the section ‘Implication of backscattered-laser measurements'.

The raw Thomson parabola (TP) data (Fig. 1c) show the dominant Al^11+^ ion and proton impurity traces along with a faint trace of Al^12+^. An atomic ionization calculation of aluminium for Trident laser parameters (*f*/3 focus) shows the aluminium is ionized to Al^11+^ and there is barely any Al^12+^ due to the huge inner-shell gap between the respective ionization potentials (Supplementary Fig. 1)^45^. The Al^11+^-ion energy spectrum measured on-axis (Fig. 1d) peaks at 166 MeV (6.2 MeV per nucleon) with a 7% energy spread. The integrated spectrum yields a total of 1.8 × 10^9^ ions per millisteradian (msr) with average energy of 123 MeV. The corresponding spectrum measured at 8.5° off-axis (Fig. 1e), for another shot with similar target and laser conditions, shows a similar ion peak at 165 MeV with 30% energy spread (total of 5.5 × 10^8^ ions per msr with average energy of 131 MeV). The proton spectrum has no spectral peak within the 9 to 50 MeV range (Supplementary Fig. 2). TP design and settings (the short gap between the electrodes and the high applied voltage) precluded measuring protons below 9 MeV. The data set associated with single-shot high-power laser systems with a long-laser cool-down period (∼1.5 h) is necessarily small. Supplementary Figure 3 shows measured off-axis Al^11+^ ion spectra for a series of five shots that illustrate the shot-to-shot variation in the results. We estimate a total of 2 × 10^11^ Al^11+^ ions in the full beam (average energy of 127 MeV; see Methods for details on conversion efficiency estimation). This result implies the aluminium ion beam contains 4 J energy out of the 80 J incident laser energy (5% conversion efficiency) and 0.35 μC of charge. A similar calculation for protons yields 0.6% conversion efficiency.

**Figure 2:**
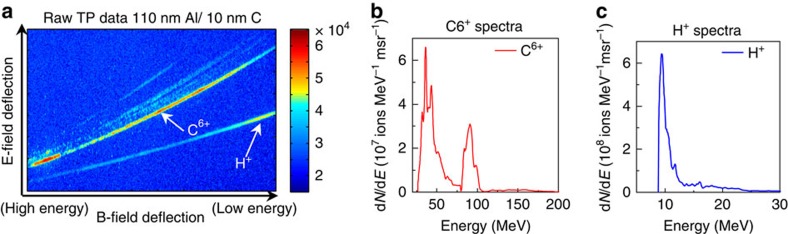
TP results from *f*/3 laser focus interaction with 10 nm carbon coated 110 nm Al foil. (**a**–**c**) (Table 1—Experiment II) (**a**) Raw TP data from 110 nm Al/10 nm C foil showing pre-dominant C^6+^ trace; (**b**) measured on-axis C^6+^ ion spectrum (solid red line); (**c**) measured on-axis proton spectrum (solid blue line).

The charge-to-mass ratios of Al^11+^ (0.407) and C^5+^ (0.417) are close. Therefore conceivably the Al^11+^ trace in the TP data could be contaminated by C^5+^ from hydrocarbon contamination of aluminium foils. To address that concern, we deliberately coated the rear side of 110-nm aluminium targets with a 10 nm of carbon layer to mimic such contamination and repeated the same experiment (Table 1—experiment II). Figure 2a shows the raw TP data from that target. The dominant trace is C^6+^, not C^5+^. Therefore, it seems likely that C, if present as a contaminant in the Al target data in Fig. 1c, then would show up as C^6+^, clearly distinguishable from the Al^11+^ trace in the TP, rather than a contributor to it as C^5+^. The C^6+^ trace in Fig. 2b from the layered target shows a spectral peak around 80 MeV (6.7 MeV per nucleon) similar to the 6.2 MeV per nucleon Al^11+^ ion spectral peak from a pure aluminium target. This suggests that the underlying laser–plasma dynamics responsible for ion spectral peak generation operate at the rear of the target, and are robust and transferable to other ion species with proper optimization. The protons again show no spectral peak above 9 MeV (Fig. 2c).

Prior experiments with a similar set-up using synthetic diamond targets^46^ did not generate quasi-monoenergetic ion beams (Table 1—experiment III)^37^ as seen here with aluminium. This difference is explained later under the section ‘Implication of backscattered-laser measurements'.

### Scaling to higher laser intensity

**Figure 3:**
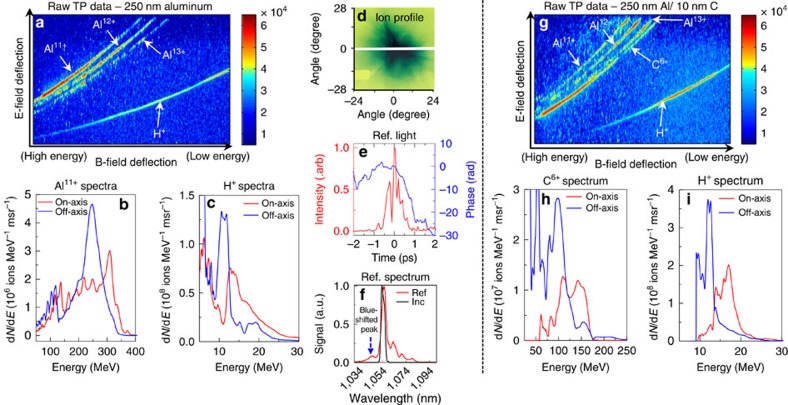
*f*/1.5 laser focus interaction with aluminium foil. (**a**) Raw TP data from *f*/1.5 laser focus interaction with the 250-nm Al foil (Table 1—Experiment IV); (**b**) spectrally peaked Al^11+^ spectra on-axis (solid red line) and 11° off-axis (solid blue line); (**c**) spectrally peaked proton spectra on-axis (solid red line) and 11° off-axis (solid blue line); **(d)** ion/proton beam profile measured using radiochromic film (RCF); (**e**) time-resolved reflected light intensity (solid red line) and temporal phase (solid blue line) indicative of relativistic transparency; (**f**) time-integrated reflected light spectrum (solid red line) along with incident light spectrum (solid black line); (**g**) raw TP data from *f*/1.5 laser focus interaction with the 10 nm carbon coated on the rear side of the 250-nm-thick Al foil (Table 1—Experiment V); **(h)** spectrally peaked C^6+^ spectra measured on-axis (solid red line) and 11° off-axis (solid blue line); **(i)** corresponding spectrally peaked proton spectra measured on-axis (solid red line) and 11° off-axis (solid blue line).

The Trident laser was focused with a faster *f*/1.5 off-axis parabola, creating a peak laser intensity of 8 × 10^20^ W cm^−2^ (4 × the *f*/3 intensity) onto a 250-nm-thick aluminium foil (Table 1—Experiment IV). The target thickness was chosen to keep the onset of relativistic transparency near the temporal peak of the laser pulse. Here two newly developed high-dispersion TPs provided simultaneous on-axis and 11° off-axis ion spectral measurements (see Methods and Supplementary Fig. 4). The raw TP data (Fig. 3a) shows well-separated traces of Al^11+^, Al^12+^, Al^13+^ and protons. The Al^11+^ trace is still dominant. The on-axis Al^11+^ ion spectrum (red solid line in Fig. 3b) peaks at 310 MeV (11.5 MeV per nucleon) with 41% energy spread (total of 4.2 × 10^8^ ions per msr with average energy of 179 MeV). The simultaneous off-axis Al^11+^ spectrum (solid blue line in Fig. 3b) peaks at 250 MeV (9.3 MeV per nucleon) with 21% energy spread (total of 4.7 × 10^8^ ions per msr with average energy of 167 MeV). The proton spectra (Fig. 3c) peaks around 12 MeV, a similar energy per nucleon as the Al^11+^.

The ion/proton beam profile (Fig. 3d) shows the ion beam extending up to a 28° FWHM. Assuming radial symmetry, we estimate a total of 1 × 10^11^ Al^11+^ ions in the full beam (average energy of 173 MeV). This implies a ∼3-J aluminium beam out of the 80 J incident laser energy (4% conversion efficiency). A similar calculation for protons yields ∼0.2% conversion efficiency. This interaction also exhibits plasma expansion towards the laser of 1.1 μm ps^−1^ derived from the reflected FROG measurement (Fig. 3e), and 1.2 μm ps^−1^ from the reflected spectral peak blue-shifted by 8 nm (Fig. 3f), similar to the results with *f*/3 and 110 nm Al.

Once again, we address the concern about possible hydrocarbon contamination of the ‘pure' Al nanofoil targets by comparing with the results from Al nanofoils coated with 10 nm of carbon layer on the rear side (Table 1—Experiment V). The raw TP data from these targets (Fig. 3g) again shows predominantly C^6+^. The C^6+^ spectrum (Fig. 3h) peaks around 120 MeV (10 MeV per nucleon) measured on-axis, and around 100 MeV (8.3 MeV per nucleon) measured off-axis, similar to the Al^11+^ energy/nucleon spectral peaks from pure aluminium targets. The proton spectrum from the carbon coated aluminium foil (Fig. 3i) peaks at 18 MeV (on-axis) and 12 MeV (off-axis).

### Implication of backscattered-laser measurements

**Figure 4:**
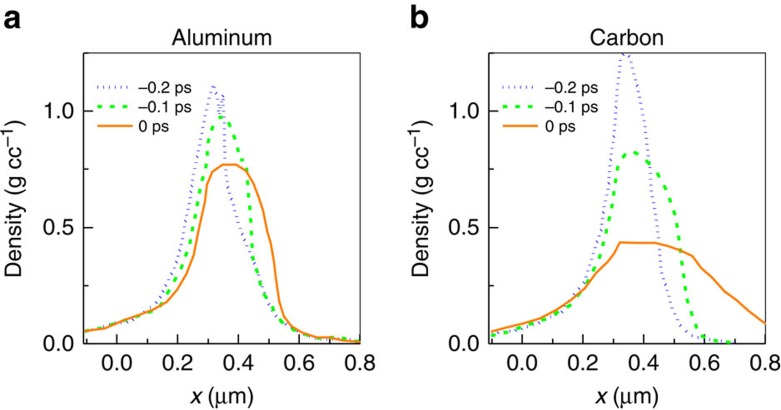
Laser pedestal pre-expansion of targets. Results from 2D rad–hydro HYDRA simulation of Trident laser pedestal (*f*/3 focus) pre-expansion of 110-nm-thick aluminium (**a**) and carbon (**b**) targets at −0.2 ps (blue dotted line), −0.1 ps (green dashed line) and 0 ps (orange solid line) before the end of the rad–hydro simulation. The targets were initially located from *x*=−0.11 μm to *x*=0 μm.

When using the same experimental set-up, the ‘Al' target produces an ion spectral peak (Table 1—Experiment I), but the ‘C' does not (Table 1—Experiment III). Here we compare the backscattered-lasers results for experiments I and III (*f*/3 laser focus interacting with 110 nm Al and 100 nm C targets, respectively) to explain the differences in their performance. A notable difference is that the backscattered-laser spectrum from ‘C' lacks a blue-shifted spectral peak (see Fig. 4b inset in ref. 44). Also, the time-dependent reflected laser from diamond (see Fig. 4b in ref. 44) shows an early frequency blue-shift of d*ω*/*ω*=0.004 (plasma expansion of 0.6 μm ps^−1^ towards the incoming laser)—nearly three times slower compared to aluminium (1.7 μm ps^−1^—Experiment I). Radiation-hydrodynamics simulations indicate, perhaps counterintuitively, that the expansion velocity during the long pedestal, which we cannot measure, is inversely correlated to the expansion velocity during the rising edge of the short-pulse, which we measure, as discussed below.

Figure 4a,b show results from two-dimensional (2D) radiation-hydrodynamics simulations of the pre-expansion of 110-nm-thick ‘Al' and ‘C' foils from the Trident laser pedestal using the HYDRA code^47^ (see Supplementary Fig. 5 for the laser contrast). The HYDRA code has been recently benchmarked against pre-expansion measurements of TNSA targets at the Trident facility^29^. Both the targets have initial density of 2.7 g cm^−3^. When the laser pedestal initially shocks the targets ‘Al' begins to disassemble faster than ‘C' (not shown). But as the shock passes through the targets ‘C' falls apart about twice as fast as ‘Al' (Fig. 4a,b). At the end of the simulations, the peak carbon density (0.4 g cm^−3^) is a factor of two lower than the peak aluminium density (0.8 g cm^−3^) when expanded by the same laser pedestal. Later, when the main pulse arrives at the plasma, it heats the plasma rapidly and consequently the plasma expansion speeds up. At the same time, the laser radiation pressure can now reduce the plasma expansion speed towards the laser (and subsequently turn it around) more effectively in carbon plasma compared with the aluminium plasma because of its lower plasma density, consistent with the backscattered FROG measurements.

Overall, these results suggest two crucial elements in our experiments that result in ion spectral peaks viz., (1) reducing pre-plasma expansion to maintain higher initial plasma density and (2) ensuring the onset of RT occurs near the peak of the laser pulse. Our hypothesis is that lower ‘initial' plasma density leads to lower residual self-generated plasma fields, too weak to reduce the ion energy spread after the laser–plasma interaction ends—supported by the particle-in-cell simulations discussed later. The above discussion was successfully put to the test by generating a quasi-monoenergetic C peak at higher intensity as discussed below.

### Optimization of laser interaction with carbon foils

**Figure 5:**
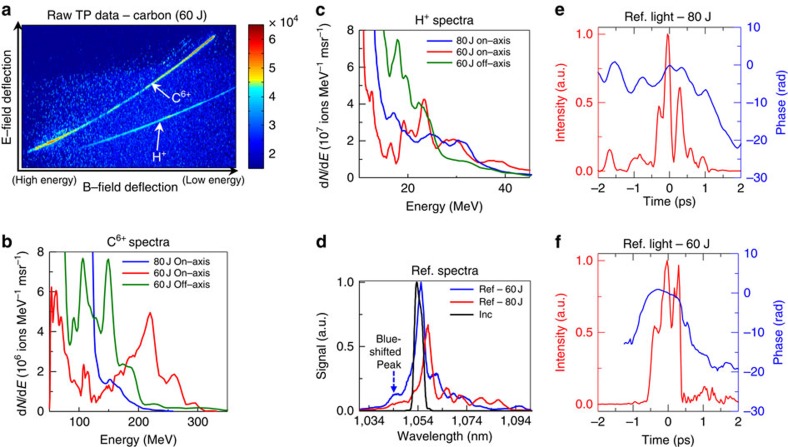
Results from *f*/1.5 laser focus onto 250 nm synthetic diamond foil. (**a**) Raw TP data from 60-J laser shot onto 250 nm diamond foil (Table 1—Experiment VII); (**b**) on-axis C^6+^ spectrum (solid red line) and off-axis C^6+^ spectrum (solid green line) from 60 J shot (Table 1—Experiment VII) showing carbon spectral peaks. On-axis C^6+^ spectrum (solid blue line) from 80-J shot (Table 1—Experiment VI) with no carbon spectral peak; (**c**) corresponding on-axis H^+^ spectrum (solid red line) and off-axis H^+^ spectrum (solid green line) from 60-J shot showing proton spectral peaks. On-axis H^+^ spectrum (solid blue line) from 80-J shot with no proton spectral peak; (**d**) reflected light spectrum from 80-J shot (solid red line) and from 60-J shot (solid blue line) along with incident laser spectrum (solid black line). Reducing the laser intensity leads to blue-shifted reflected laser spectral peak which is indicative of reduced target pre-expansion; (**e**) time-resolved reflected light intensity (solid red line) and temporal phase (solid blue line) from 80-J shot with no initial blue-shifted temporal phase indicative of significant target pre-expansion; (**f**) time-resolved reflected light intensity (solid red line) and temporal phase (dotted blue line) from 60-J shot with strong initial blue-shifted temporal phase indicative of reduced target pre-expansion.

Compared with the *f*/1.5–aluminium experiment (Table 1—Experiment IV), a similar laser interaction with a 250-nm-thick diamond foil (Table 1—Experiment VI) did not generate C^6+^ or proton spectral peaks (blue solid line in Fig. 5b,c, respectively). The early plasma dynamics of ‘C' (Table 1—Experiment VI) differ significantly from the ‘Al' (Table 1—Experiment IV)—the reflected light lacks both the initial frequency blue-shift (blue solid line in Fig. 5e) and the blue spectral peak (solid red line in Fig. 5d). Also the main reflected light peak is red shifted by 4.5 nm (solid red line in Fig. 5d), compared with only 1.7 nm for aluminium (solid red line in Fig. 3f). As above, these results indicate that the ‘C' target disassembles faster than ‘Al' during the ns laser pedestal resulting in a much lower ‘C' peak plasma density than ‘Al' when the main laser-pulse arrives. Lower plasma density enables the main laser-pulse to push the plasma away from the laser instantly and causes the lack of initial frequency blue-shift and the blue spectral peak in the backscattered-laser measurements of ‘C' that are seen in ‘Al'.

Understanding the significance of target pre-expansion, we have taken a different tack with synthetic diamond-foils to match their pre-expansion and early-expansion dynamics to that of ‘Al' foil with a view in achieving a C-ion spectral peak. We have no benchmarked way to decrease the Trident pedestal contrast any further. To reduce the diamond foil pre-expansion, we simply reduced the incident laser energy from 80 to 60 J, which correspondingly reduced the pedestal and main pulse intensities by 25% (Table 1—Experiment VII). With reduced laser energy, the reflected light shows a ‘C' foil expansion very similar to the aluminium foil interaction discussed earlier—early frequency blue-shift of d*ω*/*ω*=0.008 (solid blue line in Fig. 5f) that is a plasma expansion of 1.2 μm ps^−1^ towards the laser and a 8-nm blue-spectral peak in the reflected light (solid blue line in Fig. 5d). The corresponding raw TP data is shown in Fig. 5a. The measured on-axis C^6+^ ion spectrum (solid red line in Fig. 5b) is peaked at 220 MeV (18.3 MeV per nucleon). The simultaneous off-axis spectrum (solid green line in Fig. 5b) is double peaked at 106 MeV (8.8 MeV per nucleon) and at 150 MeV (12.5 MeV per nucleon). The corresponding proton spectrum peaks at 23.3 MeV measured on-axis (solid red line in Fig. 5c) and 17.8 MeV measured off-axis (solid green line in Fig. 5c). The same estimation as before yields a ∼4% conversion efficiency (total of 2 × 10^11^ ions, 81 MeV average energy) for C^6+^ and 0.6% for protons.

Supplementary Figure 6 shows the measured ion energy spectral peak (MeV/u) as a function of the measured plasma critical surface velocity towards the laser *v*_*cs*_ before the onset of RT for a collection of shots. The results show that the ion spectral peaks appear when *v*_*cs*_ >1 μm ps^−1^. No ion spectral peak is observed when *v*_*cs*_ <1 μm ps^−1^. Overall, these results indicate that maintaining higher initial plasma density by reducing the foil pre-expansion (thereby having a higher *v*_*cs*_ before the onset of RT) and ensuring the onset of RT occurs near the peak of the main laser-pulse are keys to generate ion spectral peaks in our experiments.

### Rad–hydro simulations of 250 nm Al target pre-expansion

**Figure 6:**
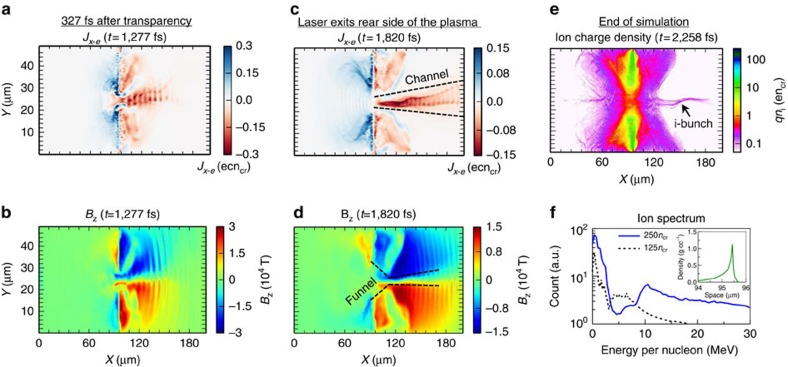
Overview of 2D VPIC simulation results from *f*/1.5 laser focus onto pre-expanded 250-nm-thick aluminium foil (**a**) Longitudinal electron current density (*J*_*x*−e_); (**b**) Self-generated azimuthal magnetic field (*B*_z_) in the plasma at *t*=1,277 fs showing the forward electron jet. Plasma transparency occurs at 950 fs; (**c**) Longitudinal electron current density (*J*_*x*−e_); (**d**) Self-generated azimuthal magnetic field (*B*_z_) in the plasma at *t*=1,820 fs when the laser exits the rear side of the plasma; (**e**) Final plasma ion charge density profile at the end of the simulation at *t*=2,258 fs with corresponding ion jet; (**f**) Final ion energy spectrum (solid blue line) with a dominant spectral peak at 10.6 MeV per nucleon; inset shows the pre-expanded plasma profile from 250-nm-thick aluminum foil that was used as input to the VPIC simulation. Dashed black line shows the ion energy spectrum with inferior spectral peak ∼5 MeV per nucleon from a simulation of non-optimal onset of relativistic transparency.

Recent study at Trident has shown that the laser pedestal pre-expansion of target must be properly accounted and provided as input to the subsequent particle-in-cell (PIC) simulation^29^. We simulate the target pre-expansion of 250-nm-thick aluminium foil (density—2.7 g cm^−3^) both in 1D and 2D using rad–hydro codes HELIOS^48^ and HYDRA^47^, respectively. The resultant 1D (Fig. 6f inset) and 2D plasma profiles (Supplementary Fig. 7) before the arrival of main pulse are exponential at the front and have a sharp drop at the rear side. The HELIOS and HYDRA results are consistent with each other. The peak density is 1.1 g cm^−3^ (that is 250*n*_cr_ for Al^11+^ ionization state). A crucial requirement for the generation of quasi-monoenergetic ion beams in our experiment is the transparency onset timing to be near the peak of the main laser pulse as indicated by the backscattered FROG measurements (Figs 1a and 3e). When the 1D pre-expanded plasma profile (Fig. 6f inset) is used as input to the subsequent PIC simulation, the onset of relativistic transparency occurs just 65 fs before the peak of the main laser pulse, consistent with experimental measurements. This qualifies the use of pre-expanded plasma profile from HELIOS as input to the ensuing PIC simulation.

### Overview of VPIC Simulations

We performed a series of 2D PIC simulations using the vector-particle-in-cell (VPIC) code^49^ to investigate the laser–plasma dynamics in the Relativistic Transparency (RT) regime with various initial target densities (125*n*_cr_ ∼ 250*n*_cr_), charge states (q/m=0.407–0.5 for heavy ions), composition and electron temperatures (1 keV ∼ 32 keV), peak laser intensities (2∼8 × 10^20^ W cm^−2^) and polarizations (*p* or *s* polarization). The collection of simulations shows the formation of a forward-propagating electron jet after the onset of relativistic transparency as a robust feature in this regime. For example, Fig. 6a and Fig. 6c show the forward electron jet at the rear of the target from the baseline simulation with 32 keV, 250*n*_cr_ target (Fig. 6f inset), heavy ion charge-to-mass ratio of 0.5, *f*/1.5 laser focus (Intensity- 8 × 10^20^ W cm^−2^), and *p*-polarized laser. Here we use the initial longitudinal density profile of a 250-nm-thick aluminium foil with laser pedestal driven pre-expansion from a 1D rad–hydro simulation, using the HELIOS code^48^ (see Fig. 6f inset), as input to the VPIC simulation. The initial plasma profile is exponential at the front side and peaks at *x*=95.4 μm with a peak density of *n*_e_=250*n*_*cr*_. On the rear side, the profile drops sharply in 0.27 μm (see Methods for further details). Key laser time markers in the simulation are: laser is launched from the left boundary at *t*=0 fs; laser reaches target at *t*=315 fs; onset of transparency occurs at *t*=950 fs; laser exits the rear side of the plasma at *t*=1,785 fs; laser exits the right boundary at *t*=2,100 fs; and the simulations ends at *t*=2,258 fs. The laser pulse full duration in the simulation is 1,400 fs.

The electron jet emerges shortly after the onset of RT, then extends in the forward direction as shown in Fig. 6a at 327 fs after RT, and finally forms a long channel over ∼100 μm as the laser exits the plasma (Fig. 6c for *t*=1,820 fs). The magnitude of the longitudinal current density in the jet is *J*_*x*−e_ ≈ *en*_e_*c* ∼ *en*_cr_*c*. Assuming a radius comparable to the laser spot size ‘*D'*, the jet carries an enormous current ∼π(*D*/*λ*)^2^ (*n*_*e*_/*n*_*cr*_)^2^*I*_A_ and the maximum of the associated quasi-static azimuthal magnetic field is ∼(*D*/*λ*)*B*_0_ at the outer radius of the jet (Fig. 6b,d). Here ‘*λ'* is the laser wavelength, *I*_A_≈17 kA is the Alfvén current and *B*_0_ =*mcω*_0_/*e*≈1.02 × 10^4^ tesla. The magnetic field has the shape of a ‘funnel' and it is strongest at ∼20−30 μm ahead of the initial target position, decreasing gradually further away as the jet diverges. The quasi-static magnetic field persists for >400 fs after the laser exits the plasma. Supplementary Movie 1 shows the evolution of the self-generated azimuthal magnetic field for the full duration of the VPIC simulation. Although self-generated magnetic fields have been reported in previous studies of laser-driven plasmas^39^,^40^,^41^,^50^,^51^,^52^,^53^,^54^,^55^, they have not been associated with reducing the ion energy spread as discussed below.

Despite their much smaller charge-to-mass ratio, the plasma ions essentially follow the electron motion and form a similar jet (Fig. 6e). Supplementary Figure 8 shows the corresponding electron-charge density snapshot at the end of the simulation. The ion energy spectrum within the plasma channel at *t*=1,820 fs when the laser exits the rear side of the plasma is still exponential (not shown). Also, we remark for later that the ion jet is chirped in energy along the channel—the ions with the highest (lowest) energies are leading (lagging). By *t*=2,258 fs, the simulation shows a 10.6 MeV per nucleon ion spectral peak (Fig. 6f solid blue line) consistent with our experiments. The black arrow in Fig. 6e indicates the location of the 10.6 MeV per nucleon spectral peak in the ion jet. The spectrum in Fig. 6f also shows another minor ion spectral peak at 1 MeV per nucleon.

The formation of the ion spectral peak as seen in the VPIC simulation is the result of an integrated, self-consistent dynamical process lasting few hundred femtoseconds after the laser exits the rear side of the plasma. To grasp it, we break those dynamics during that period into three separate parts: the electron dynamics, the electrostatic-field dynamics, and the ion dynamics. The magnetic field and the plasma channel that it defines, although part of the self-consistent dynamics, evolve on longer timescales and are therefore considered as given for the sake of understanding.

### Late-time electron injection and slow down

**Figure 7:**
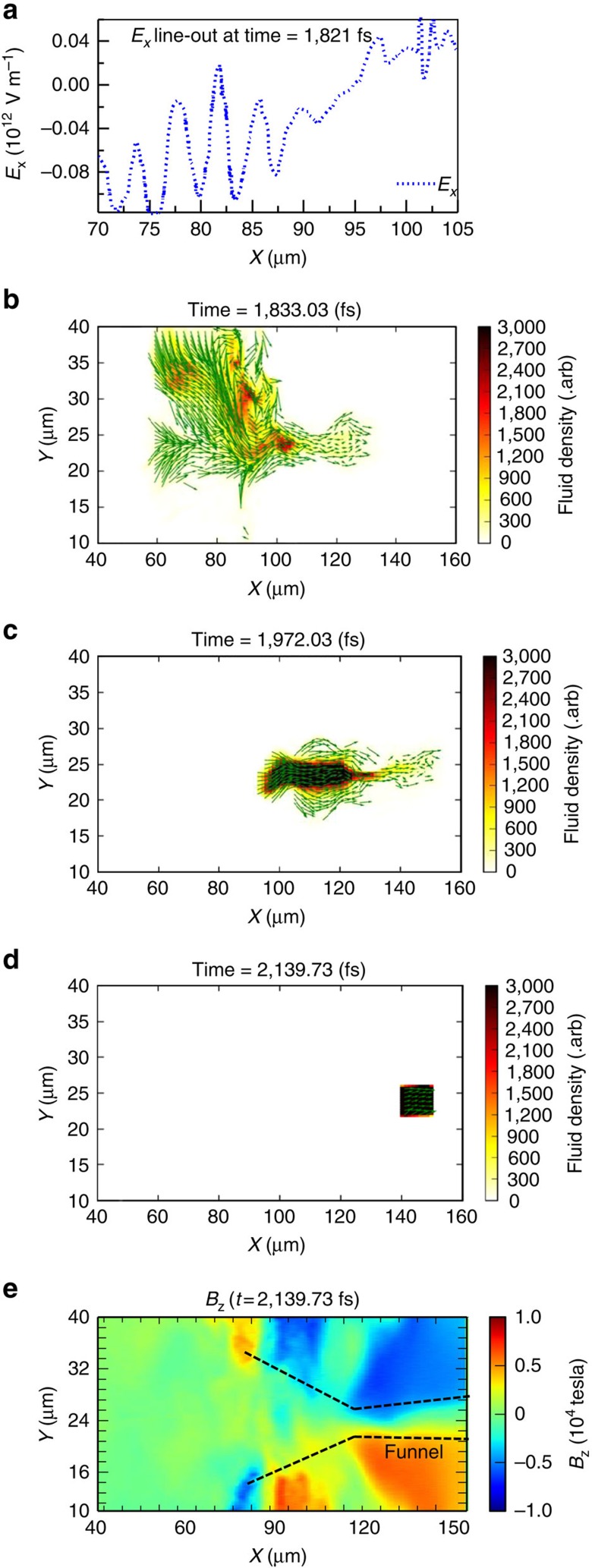
Electron injection into the plasma channel, and its subsequent slow down after the laser exits the plasma. (**a**) Longitudinal *E*_*x*_ line-out along the middle of the simulation box (averaged over 21 μm<*y*<27 μm) at *t*=1,821 fs responsible for electron injection into the plasma channel. (**b**–**d**) three snapshots of the spatial distribution and the fluid velocity vectors of the tracked electron macroparticles at *t*=1,833 fs, 1,972 fs, and 2,140 fs, respectively. The length of the fluid velocity vectors is proportional to fluid velocity. The electrons are selected from a rectangular region 140 μm<*x*<150 μm and 21 μm<*y*<25 μm at *t*=2,142 fs just ahead of the ion spectral peak location; (**e**) self-generated azimuthal magnetic field (*B*_z_) at *t*=2,140 fs long after the laser is gone.

We track the electron macroparticles located immediately ahead of the 10.6 MeV per nucleon ion spectral peak near the end of the simulation (Fig. 7d) backward in time. This is important because these electrons are a critical component of the charge density profile inside the plasma channel that leads to the electric field that in turn rotates the ion phase space distribution that shapes the ion peak of interest. The Eulerian fluid velocity vector and the spatial density distribution of the tracked macroparticles are shown for three stages of their motion: the injection at the front side (*x*≈60–90 μm), the slow down in the mouth of the magnetic funnel (*x*≈90–120 μm) and further slow down of the localized density peak inside the channel (around *x*=140 μm). Representative snapshots of these stages (*t*=1,833, 1,972 and 2,140 fs) are shown in Fig. 7b–d (see Supplementary Movie 2).

The injection of electrons is a result of the sheath field at the front side of the target, shown in Fig. 7a for *t*=1,821 fs, shortly after the laser exits the target. At this stage, these electrons are spatially dispersed but mostly come from the front side of the target (see Fig. 7b for their distribution at *t*=1,833 fs). They are accelerated forward to ∼1−3 MeV in the sheath potential at the front side of the target and injected into the mouth of the magnetic funnel (*x*≈90–120 μm) and further down through the channel (*x*>120 μm; also see Fig. 6c). Those electrons that encounter the large azimuthal magnetic field near the mouth of the funnel (*x*≈90–120 μm) become strongly magnetized with their gyro-radii on the order of a few microns.

The transverse and the longitudinal magnetic field gradients there significantly slow down the electron fluid drift speed (the cyclotron averaged motion of the ensemble) but not the speed of the individual electrons. Hence, at this compression stage, the slow down of the electron fluid increases the electron density locally^56^. (The electrostatic force plays a negligible role here.) However, this barrier is weakest near the channel axis where the magnetic field and its gradient are smallest and the electrons stream through the channel (Fig. 7c).

**Figure 8:**
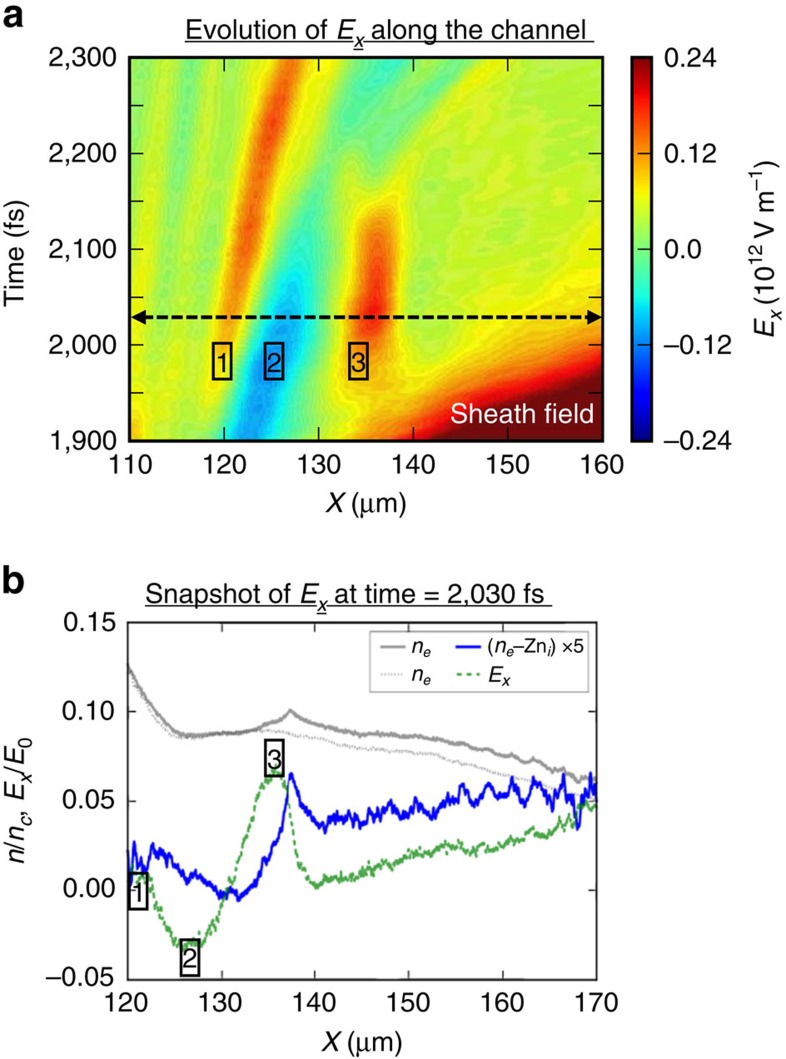
Longitudinal electric field along the plasma channel responsible for ion energy spread reduction. (**a**) late-time evolution of the longitudinal electric-field line-out (averaged over 21 μm<*y*<27 μm) along the channel from *t*=1,900 fs to *t*=2,300 fs after the laser exits the plasma; (**b**) The electron (solid grey line), ion (dotted grey line), and net charge (solid blue line) densities and the resulting longitudinal electric field (dashed green line) along the plasma channel at *t*=2,030 fs that leads to the particular electric field pattern along the plasma channel.

In the third stage, these electrons overtake the ions of interest (the ions that will form a spectral peak) and encounter another magnetic barrier, a kink in the channel around *x*=135 μm. Around that point, the electron forward motion slows down and their density increases again. The resulting electron-density peak causes an electric field labelled ‘3' in Fig. 8 (discussed below).

### Late-time evolution of the longitudinal electric field

The dynamics described above leads to a double-hump longitudinal electric-field pattern (Fig. 8a) along the plasma channel after the passage of the detached sheath field driven by the laser. Figure 8b shows the line-outs of the ion, electron and net charge densities together with the longitudinal electric field along the plasma channel at *t*=2,030 fs (marked by dashed black line in Fig. 8a). The first electric-field peak (labelled ‘1' in Fig. 8a) results from the slow down of the electron fluid near the ‘mouth' of the magnetic ‘funnel' increasing the electron density locally around *x*=120 μm (Fig. 8b). The excess electron-charge density decreases around *x*=125 μm (Fig. 8b) that causes the electric-field valley (labelled ‘2' in Fig. 8a).

The near-stationary transient electric-field peak inside the plasma channel between *t* ∼1,950 fs and *t* ∼ 2,150 fs (labelled ‘3' in Fig. 8a around *x*=135 μm) is associated with the nearby electron-density peak (Fig. 8b). It is important to realize that the electron fluid inside the plasma channel is in a continuous motion and it does not stand still anywhere inside the plasma channel as seen in the Supplementary Movie 2. Rather, a decrease in the magnetized electron fluid velocity around *x*=135 μm increases its density at that position^56^.

The double-hump electric-field pattern is sustained by the continuous injection of electrons from the front side of the plasma. As the injected electron current weakens over time, the magnetic field also weakens and recedes away from the target (see Supplementary Movie 1). The phase velocities of the first peak/valley are synchronous with the phase velocity of the magnetic-field recession, which is a small fraction of the speed of the light and close to the background ion velocity.

### Ion spectral peak formation

**Figure 9:**
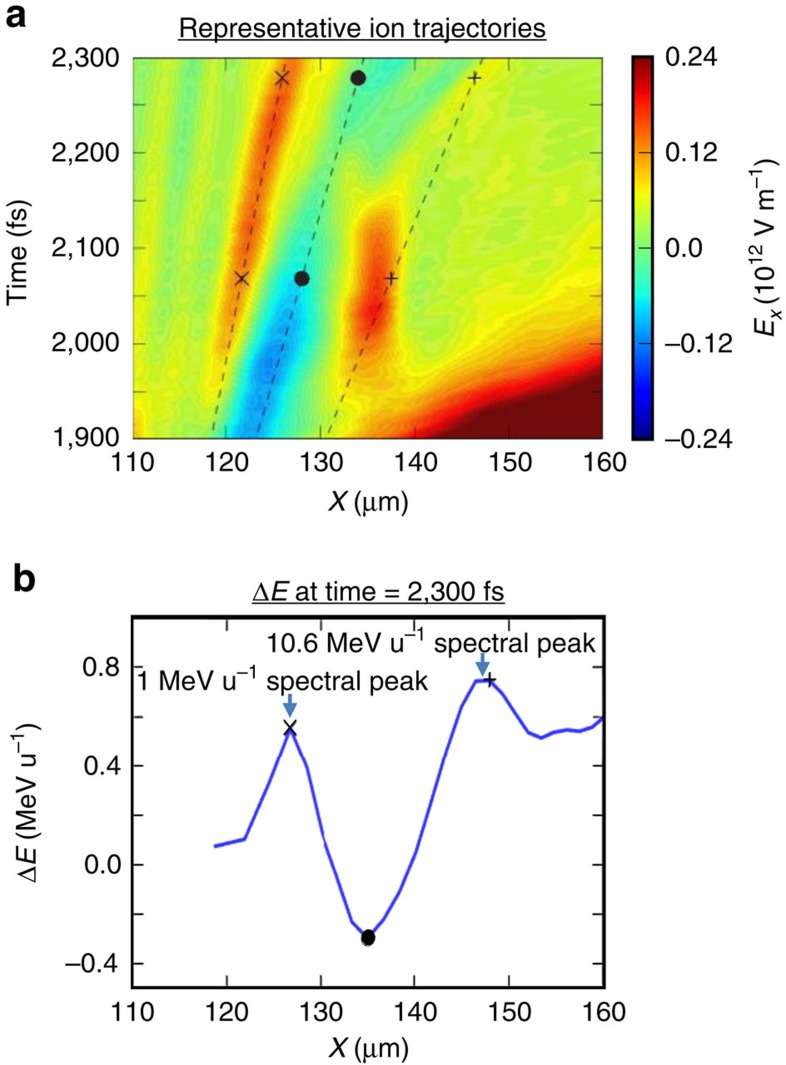
Ion energy spread reduction from the longitudinal electric field after the laser exits the plasma. (**a**) Three representative ion trajectories (marked by × , ·, +) overlaid on top of the evolving longitudinal electric field in the plasma channel; (**b**) energy gain/loss per nucleon at *t*=2,300 fs with respect to the ion location in the plasma channel at *t*=1,900 fs. Also marked is the final energy gain/loss of the representative ions in the spectral peaks.

Figure 9 shows representative trajectories of ions that traverse the plasma channel and the associated late-time electric-field pattern between *t* =1,900 fs and *t* =2,300 fs and their energy gain/loss. As the ion beam has an energy chirp (increasing from left to right), both the slow-moving and the nearly stationary transient longitudinal electric fields can generate ion spectral peaks. The representative ion with trajectory marked by ‘ × ' (‘·') in Fig. 9a has lower (higher) initial energy due to the energy chirp and moves synchronously with the first electric-field peak (valley). This causes phase space rotation, forming an ion spectral peak. Another spectral peak is formed by the stationary electric field (labelled ‘3') as it has finite time duration and is temporally separated from other field patterns (the earlier sheath field that moves away quickly and the field valley, labelled as ‘2', that moves in at a later time). The energy gain of the ion is correlated with its arrival time at the location of this transient field. Those fast (slow) ions arrive too early (late) with respect to the field duration do not gain energy, while those ions that arrive at the right time and have transit times across the field smaller than the field duration will gain the same energy. Therefore a spectral peak will form due to the variation in energy gain correlated with the energy chirp of the ions. Figure 9b shows the ion energy gain/loss per nucleon between *t*=1,900 fs and *t*=2,300 fs with respect to their location in the plasma channel. This result shows spectral peaks at 1 MeV per nucleon and 10.6 MeV per nucleon from the slow moving E-fields and from the near-stationary electric field, respectively. The 10.6 MeV per nucleon peak is close to the experimental results. No persistent ion spectral peak at higher energy is formed from the detached sheath field that moves substantially faster than the ions, despite its much larger amplitude.

Our rad–hydro simulation indicates that ‘C' foil expands more than the ‘Al' foil during the laser-pulse pedestal for the same laser parameters, resulting in lower initial peak plasma density that leads to non-optimal onset of relativistic transparency and lack of ion spectral peak. This effect is investigated in the simulation with a lower initial plasma density (a peak plasma density of 125 *n*_cr_) but other parameters are kept the same. While the essential processes discussed above are present in this simulation, the onset of relativistic transparency for this target occurred at 270 fs earlier than for the 250 *n*_cr_ target, leading to weaker electron jet and residual self-generated fields (∼1/3 of those in the 250*n*_cr_ simulation). The resulting ion energy spectrum (dashed black line in Fig. 6f) shows a less pronounced ion spectral peak at 5 MeV per nucleon. Therefore, it is likely that the target pre-expansion plays an important role both in setting up strong self-generated plasma fields and relativistic transparency onset timing leading to the subsequent ion spectral peak formation.

## Discussion

The question arises as to whether the ion spectral peaking reported here could be due to a different mechanism, such as magnetic vortex acceleration as defined in ref. 42. ‘Magnetic vortex acceleration' relies on self-generated quasi-static magnetic field at the rear side of the plasma for efficient forward ion acceleration and collimation^39^,^40^,^41^,^42^. In the magnetic vortex mechanism, as explained in ref. 42, magnetic pressure expels electrons from the magnetic region into the plasma channel and builds up an electrostatic field, which accelerates the ions forward at the plasma–vacuum interface^42^. In our case, we also see self-generated quasi-static magnetic field at the rear side of the plasma similar in nature to ref. 42. However, in our case the electrons responsible for reducing the ion energy spread are injected from the front side of the plasma and not from the magnetic field surrounding the plasma channel. Several other concerns and issues such as contribution of hole-boring radiation-pressure ion acceleration, tamping of heavy ions by protons, origin of proton spectral peaks, and the relevance to BOA mechanism are discussed in detail under the section ‘Supplementary Discussion' in the Supplementary Information.

In summary, we have demonstrated laser-driven ion beams with narrow spectral peaks at energies up to 18 MeV per nucleon and ∼5% conversion efficiency from 0.12 PW laser interactions with planar foils. Computer simulations show a self-organizing scheme that reduces the ion energy spread using self-generated fields from optimized laser–plasma interactions in the relativistic transparency regime. Furthermore, divergence control (collimation/focusing) of these energetic narrow energy spread ion beams is a crucial next step in making these ion beams suitable for various applications^57^,^58^,^59^,^60^. Also, a lot of underlying microphysics still remains to be understood in detail. For example, these include identifying the exact relationship between the target pre-expansion and the amount of electron current/associated magnetic field, how exactly the timing on the onset of relativistic transparency affects the ion energy spread reduction, and relevance/consequence of Alfvén limit in relativistic laser–plasmas^61^.

## Methods

### Laser system and ion diagnostics

The experiments were conducted at Trident laser facility at Los Alamos National Laboratory, USA. The Trident laser (80 J, 650 fs FWHM, 1,053 nm wavelength, linear polarization) is focused at normal incidence onto the target using an *f*/3 off-axis parabola (OAP) to a spot size of 10 μm diameter (first Airy minimum containing 65% laser energy) with a peak laser intensity of 2 × 10^20^ W cm^−2^ (*a*_0_≈13). Plasma mirrors were not used in the experiment. A high resolution TP employing 0.91 T magnetic field over 20-cm long and a pair of copper electrodes, also 20-cm long, charged up to 28 kV potential was used to quantify the resulting ion spectra from the laser–plasma interaction at on-axis (0 degrees) and off-axis (8.5 degrees)^62^. Image plates were used as ion detectors in the TP and they were cross-calibrated against CR-39 nuclear track detectors. The instrumental cut-off is at 50 MeV for Aluminium ions due to an 18-μm-thick aluminium foil placed in front of the ion detector to block laser light. The protons had a low energy cut-off at 9 MeV due to the size of the electrodes and the image plate. For this particular experiment the TP was rotated between on-axis and off-axis for ion spectra characterization. The divergence of the ion beam profile was characterized using a magnetic spectrometer called iWASP (ion wide angle spectrometer)^63^ and image plate detectors. The iWASP was used only on selected shots as it would block the use of any diagnostic located further downstream.

The second set of experiments used a faster *f*/1.5 OAP to focus the same Trident laser to a spot size of 5-μm diameter (first Airy minimum containing 65% laser energy) with a peak laser intensity of 8 × 10^20^ W cm^−2^. For *f*/1.5 experiments we developed two additional high-dispersion Thomson parabolas employing longer electrodes (up to 50 cm), shorter magnetic field (0.82 tesla over 10-cm long), and longer drift length that enabled better separation of traces with different charge states. The two TPs were located side-by-side on-axis and 11° off-axis enabling the simultaneous on-axis and off-axis TP data collection (see Supplementary Fig. 4 for more details). The maximum raw signal strength in the TP data reported in this article was 5.7 × 10^4^, which is well below the saturation limit of 6.5 × 10^4^. The ion beam profile in this experiment was measured using a radiochromic film (25 × 20 cm with 1-cm gap in the middle for downstream diagnostics) instead of the iWASP magnetic spectrometer used earlier. The front of the RCF was covered with 75-μm-thick aluminium foil to block the laser light, aluminium ions up to 180 MeV and protons up to 3 MeV. Although we cannot separate the ion and proton beam profiles using the RCF signal in our configuration, we assume they have similar divergence as shown by the earlier iWASP measurements in Fig. 1g.

### Conversion efficiency estimation

For example, the conversion efficiency from laser energy to aluminium ions in our experiment is estimated as follows. First, we integrate the aluminium ion energy spectra from the TP in Fig. 1d,e to obtain the aluminium ion yield per unit solid angle at 0° and 8.5°, respectively. The angularly resolved ion energy spectrum along the horizontal plane with iWASP spectrometer in Fig. 1g shows aluminium ions up to 17° half-width-half-maximum. We integrate the measured ion beam profile along this horizontal slice by using the 0° and 8.5° ion yields as anchors to enable an interpolation of the ion yields/solid angle for the full angular distribution. Assuming radial symmetry, the total ion yield is computed from the interpolated angular distribution. Previous measurements at the Trident laser facility in the relativistic transparency regime using diamond targets have shown that the ion yields are almost independent of whether the measurement is made in the horizontal plane or in the vertical plane^46^. On the basis of this result, we expect that the measured aluminium ion spectra in Fig. 1d,e may be assumed to be radially symmetric.

### Particle-in-cell simulation

Two-dimensional VPIC^49^ simulations were performed with the Trident laser parameters. The simulation box size is 200 μm in *x* direction (laser propagates from left to right) and 50 μm in *y* direction with an exponential aluminium plasma profile facing the incoming laser. We used 382 cells per wavelength in the *x* direction and 256 cells per wavelength in the *y* direction in the simulation. In the nominal simulation discussed in the article, the slab target consists of electrons and fully ionized ions with a charge-to-mass ratio of 0.5*e*/*m*_*p*_, where *m*_*p*_ is the proton mass. This charge-to-mass ratio is the same as C^6+^ ion and close to the Al^11+^ ion, the dominant ion species found in the experiments using diamond-like carbon and aluminium foil targets, respectively. We used 625 particles per cell for each species in the simulation. Initial target temperatures are *T*_*e*_=32 keV and *T*_*i*_=1 keV, respectively. The *p*-polarized laser at 1 μm wavelength is launched from the left boundary of the box and focused at the peak target density at *x*=95.4 μm. The laser intensity has a temporal profile of *I*(*t*)=*I*_peak_sin^2^(*πt*/2*τ*), where *τ*=650 fs (The laser pulse full duration is 1,400 fs that is 420-μm long in free-space). The transverse laser intensity profile is Gaussian with a spot size of 2.5 μm (*f*/1.5 laser focus) and the peak intensity is *I*_peak_=8 × 10^20^ W cm^−2^ (*a*_*0*_=24). The particles were injected into the simulation box right before the laser reaches the target front surface to avoid plasma self-expansion.

## Additional information

**How to cite this article:** Palaniyappan, S. *et al.* Efficient quasi-monoenergetic ion beams from laser-driven relativistic plasmas. *Nat. Commun.* 6:10170 doi: 10.1038/ncomms10170 (2015).

## Supplementary Material

Supplementary Information Supplementary Figures 1-8, Supplementary Discussion and Supplementary References.

Supplementary Movie 1 Supplementary video 1 shows the evolution of self-generated plasma magnetic field (Bz) from the VPIC simulation with Trident laser interacting with 250ncr plasma. The laser has a Full-Width-Half-Maximum of 650 fs and full duration of 1400 fs (i.e., 400 optical cycles). The time-stamp in the video is in units of laser optical cycle i.e., 3.5 fs. The laser is launched 5 microns away from the left boundary at t=0 fs. Coming from the left boundary, the laser takes 315 fs (~90 optical cycles) to reach the target placed at 95 ?m. At t = 1785fs (~510 optical cycles) the trailing edge of the laser begins to exit the plasma. The simulation ends at t=2258fs (645 optical cycles).

Supplementary Movie 2 Supplementary video 2 tracks the electron bunch associated with the 10.6 MeV/nucleon ion bunch. These particles were selected and tagged at the end of the simulation. After tagging the simulation was re-run from t = 1833 fs to t = 2140 fs. The video shows the electron injection, slow down and localization after the laser exits the plasma. If there is any other editorial revision that is required or something that we missed we will be happy to make the necessary changes to the manuscript. Finally we would like to thank the editor and the referees for a fair, efficient and thoughtful review of our manuscript

## Figures and Tables

**Table 1:** Laser/target parameters and properties of generated ion beams and plasmas.

**Experiment.**	**Laser focus**	**Intensity (W cm**^−2^)	Target*	***v***_***cs***_**(μm ps**^−1^)†	**Ion spectral peak**‡	**FWHM Energy spread**‡ **(%)**	**Conversion efficiency (%)**	**Proton spectral peak**‡ **(MeV)**
I	*f*/3	2 × 10^20^	110 nm Al	1.7	165 MeV Al^11+^ (6.1 MeV/u)	7	5	Below TP cut-off
II	*f*/3	2 × 10^20^	110 nm Al/10 nm C	−	80 MeV C^6+^ (6.7 MeV/u)	15	−	Below-TP cut-off
III	*f*/3	2 × 10^20^	100 nm C	0.6§	No spectral peak||	−	−	No spectral peak||
IV	*f*/1.5	8 × 10^20^	250 nm Al	1.1	310 MeV Al^11+^ (11.5 MeV/u)	41	4	12.6
V	*f*/1.5	8 × 10^20^	250 nm Al/10 nm C	−	120 MeV C^6+^ (10 MeV/u)	54	−	17
VI	*f*/1.5	8 × 10^20^	250 nm C	−0.46	No spectral peak	−	−	No spectral peak
VII	*f*/1.5	6 × 10^20^	250 nm C	1.2	220 MeV C^6+^ (18.3 MeV/u)	23	4	23.3

FWHM, full width at half maximum; TP, Thomson parabola ion energy diagnostic; *v*_*cs*_, plasma expansion speed towards laser before the onset of relativistic transparency.

^*^‘a/b' notation means the target ‘b' is coated on the rear side (away from the incoming laser) of target ‘a'.

^†^Obtained from the frequency shift of the laser light reflected from the plasma before the onset of relativistic transparency using single-shot FROG (frequency-resolved-optical-gating).

^‡^Measured at 0° on-axis with the laser beam; first number denotes ion spectral peak. Number in parenthesis denotes the same in units of energy per nucleon.

^§^Obtained from ref. 44.

^||^Obtained from ref. 37; — not discussed in the article.
