# Supplementary material for: Efficient quasi-monoenergetic ion beams from laser-driven relativistic plasmas
Source: Nat Commun. 2015 Dec 11;6:10170. doi: 10.1038/ncomms10170 (PMC4682178; doi:10.1038/ncomms10170)
Supplement: Supplementary Information — Supplementary Figures 1-8, Supplementary Discussion and Supplementary References. [file ncomms10170-s1.pdf]

Atomic ionization of aluminum

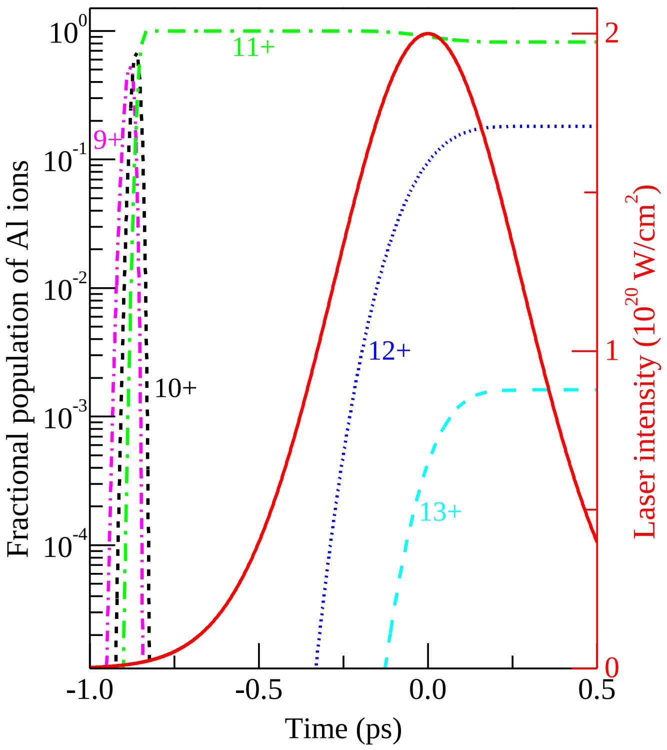

Supplementary Figure 1: Laser field ionization of aluminum atom by the Trident laser pulse The figure above shows the fractional population of aluminum ions when Trident laser pulse ionizes an aluminum atom. This is calculated using Ammosov-Delone-Krainov (ADK) tunneling ionization rate (Ammosov, M.V., N.B. Delone, and V.P. Krainov, *Tunneling ionization of complex atoms and of atomic ions in an alternating electric field.* Sov. Phys. JETP, 1986. **64**: p. 1191-1194). The result shows that almost the entire aluminum atom becomes aluminum 11+ ion at the foot of the laser pulse (the ionization potential to strip an electron from Al^{10+} to get Al^{11+} is 442 eV). The populations of aluminum 12+ and 13+ ions begin to increase only near the peak of the laser pulse due to larger *K*-shell ionization gap of 1644 eV (the ionization potential to strip an electron from Al^{11+} to get Al^{12+} is 2086 eV). The populations of Al^{11+}, Al^{12+} and Al^{13+} ions remain at a fixed level of 81.7%, 18.1% and 0.2% for most of the laser pulse duration. Since the peak intensity is reached only at the middle of the focus, in the Thomson parabola we should

expect to see mostly Al^{11+} ions along with very little Al^{12+} from the Trident laser pulse interaction with aluminum.

Proton Spectra

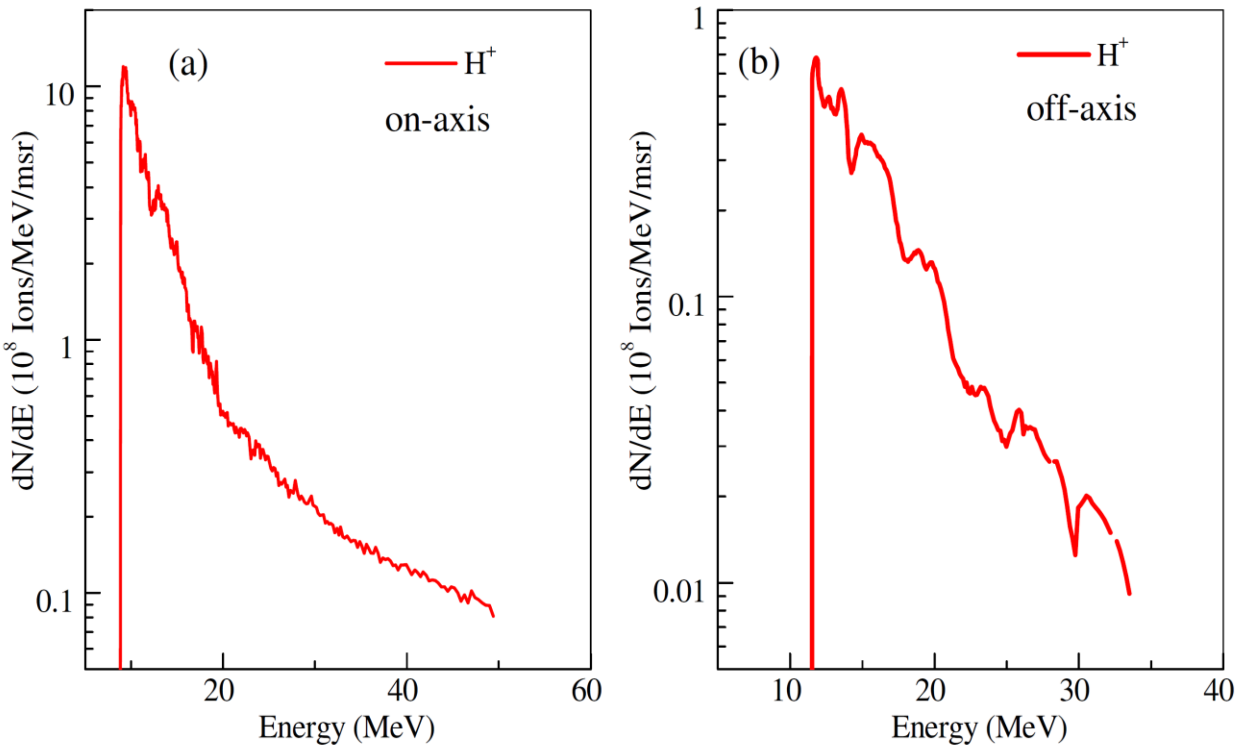

Supplementary Figure 2: Proton energy spectra Supplementary figure 2 (a&b) show the proton energy spectra from Trident laser (peak intensity 2 x 10^{20} W/cm^2) interacting with 110 nm thick aluminum foil measured on-axis (a) and 8.5° off-axis (b). The spectral shape is exponential above the instrumental cut-off at 9 MeV. Calculation shows 4 x 10^9 protons per millisteradian on-axis and 2.8 x10^8 protons per millisteradian off-axis with average energy of 13 MeV. Estimation of the total number of protons with a methodology similar to the one used for Al^{11+} in the manuscript yields a total of 2.3 x 10^{11} protons corresponding to proton conversion efficiency of 0.6%.

Al^{11+} ion energy spectra

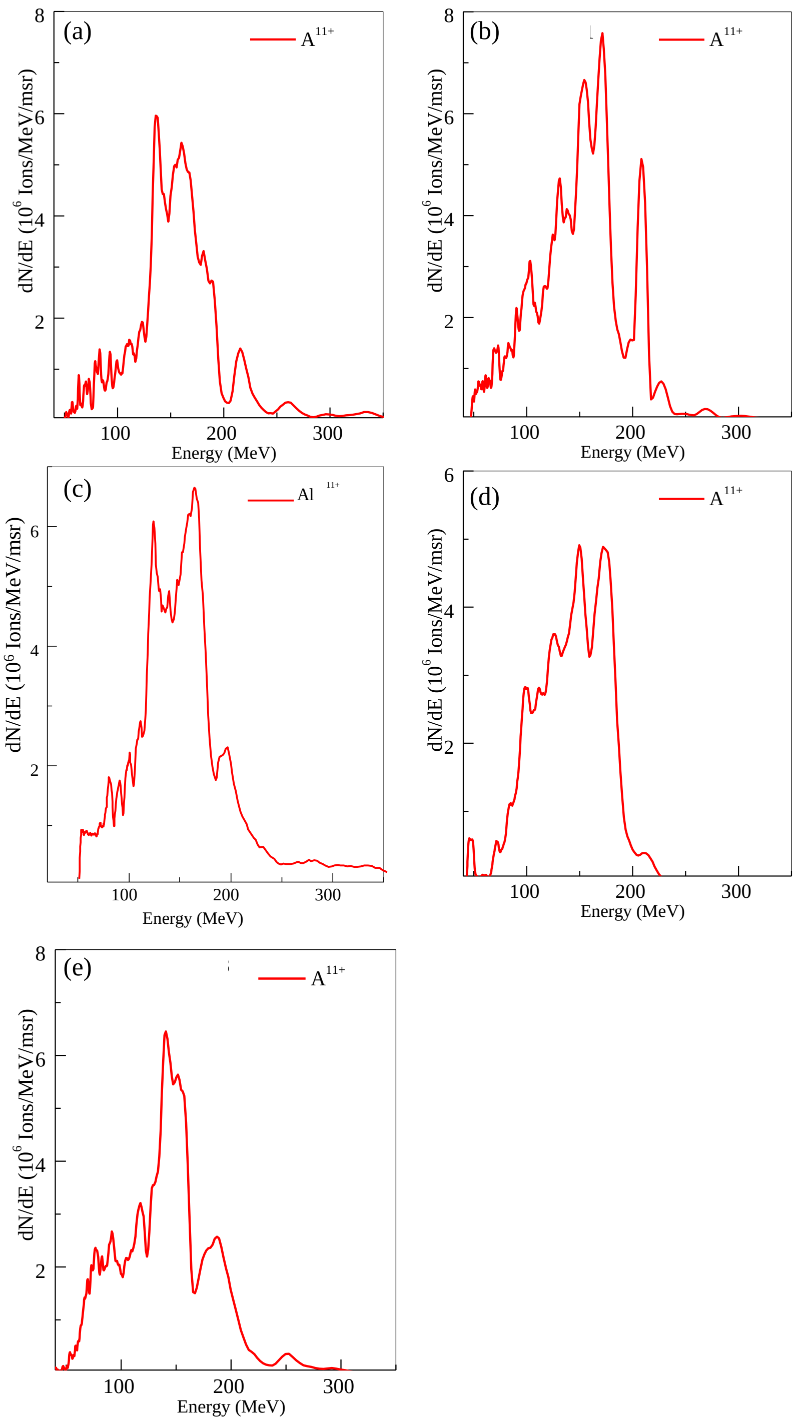

Supplementary Figure 3: Al^{11+} ion energy spectra for a series of five shots The figure above shows the measured off-axis Al^{11+} ion energy spectra for a series of five shots. These shots show

ion spectral peaks around 150 MeV. The data set associated with single-shot high power laser systems is small by their nature. Together they represent the shot-to-shot variation in the results.

High-dispersion Thomson Parabola

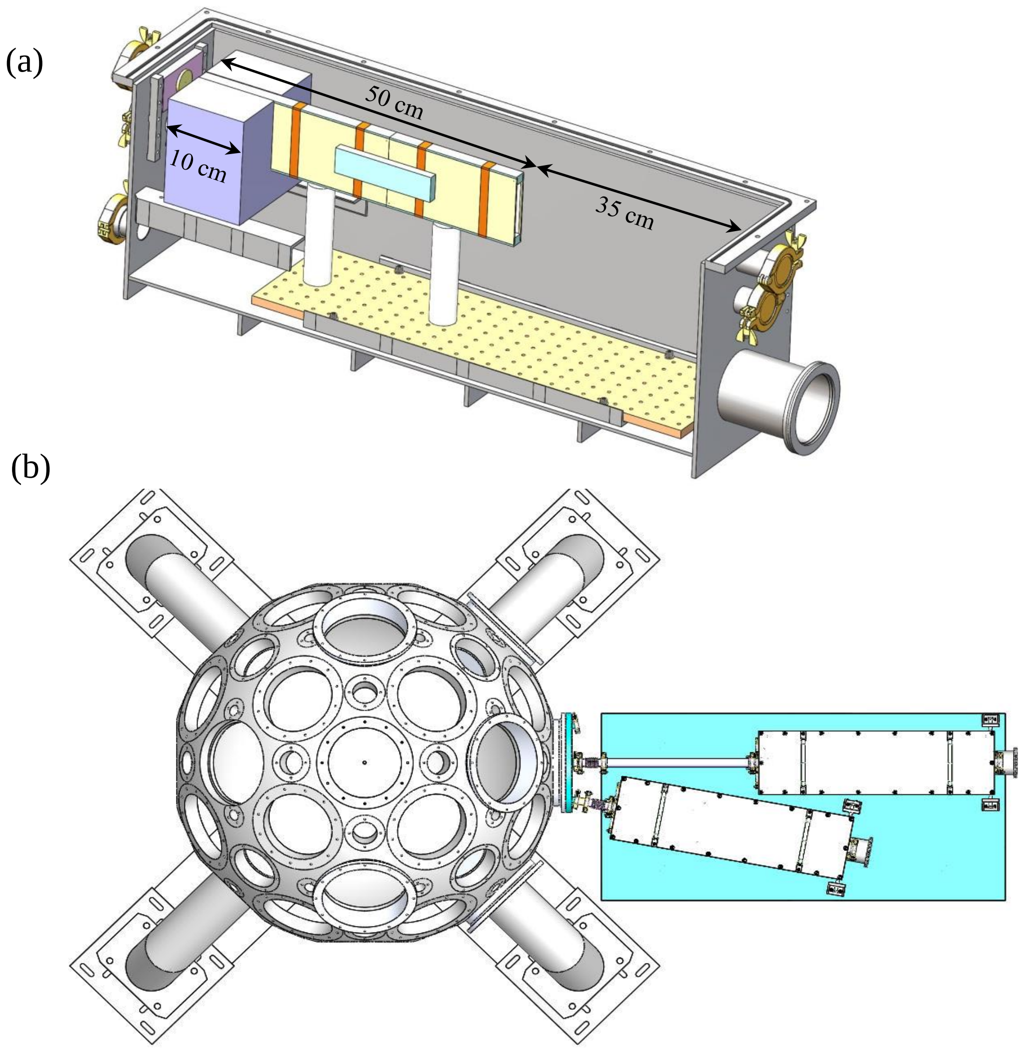

Supplementary Figure 4: High dispersion Thomson Parabola Supplementary figure 4a shows the side-view of the high-dispersion Thomson parabola without the front panel for better visibility. The TP uses 0.82 Tesla magnets (10 cm long) and 50 cm long electrodes that can be charged up to 15kV potential. The drift length from the end of the electrode to detector (placed at the end of the box) is 35 cm. Supplementary figure 4b shows the two TPs stacked side-by-side

next to the Trident north target chamber. The two TPs simultaneously measured the on-axis and 11° off-axis ion spectra.

Trident Laser Contrast

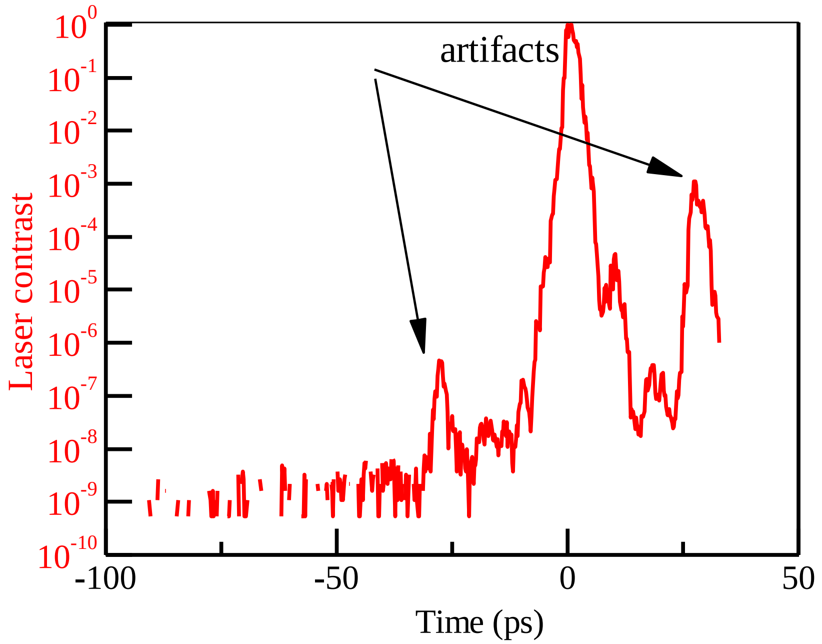

Supplementary Figure 5: Trident laser contrast Supplementary figure 5 shows the Trident laser contrast measured using a scanning third order auto-correlator (Rincon, DelMar Photonics). The laser pedestal drops to 10^{-8} nearly 10 ps before the laser peak. The detection limit is 10^{-9}. The two peaks at ±27 ps are measurement artifacts from the scanning device.

Correlation between critical surface velocity and Ion spectral peak

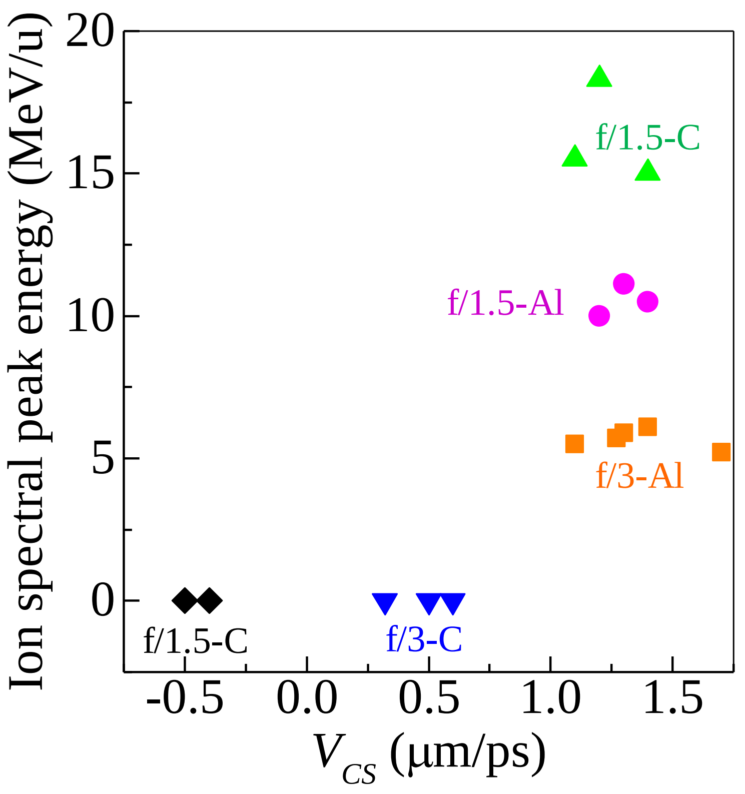

Supplementary Figure 6: Critical surface velocity Vs ion spectral peak Supplementary Figure 6 shows the observed ion spectral peak energy/nucleon as a function of plasma critical surface velocity towards the laser 'v_{cs}' before the onset of relativistic transparency for a collection of 16 shots. The notation 'f/1.5-C' for example means f/1.5 laser focus interaction with 'C' target. The result shows that ion spectral peaks appear when 'v_{cs}' is greater than 1 μm/ps. No ion spectral peaks are observed when 'v_{cs}' is less than 1μm/ps (dots along the zero energy/nucleon line). Overall, these results indicate that maintaining higher initial plasma density by reducing the foil pre-expansion (which leads to higher critical surface velocity towards the laser) and ensuring the onset of relativistic transparency occurs near the peak of the main laser pulse are keys to reducing the laser-driven ion energy spread.

250 nm Aluminum foil target pre-expansion using 2D HYDRA code

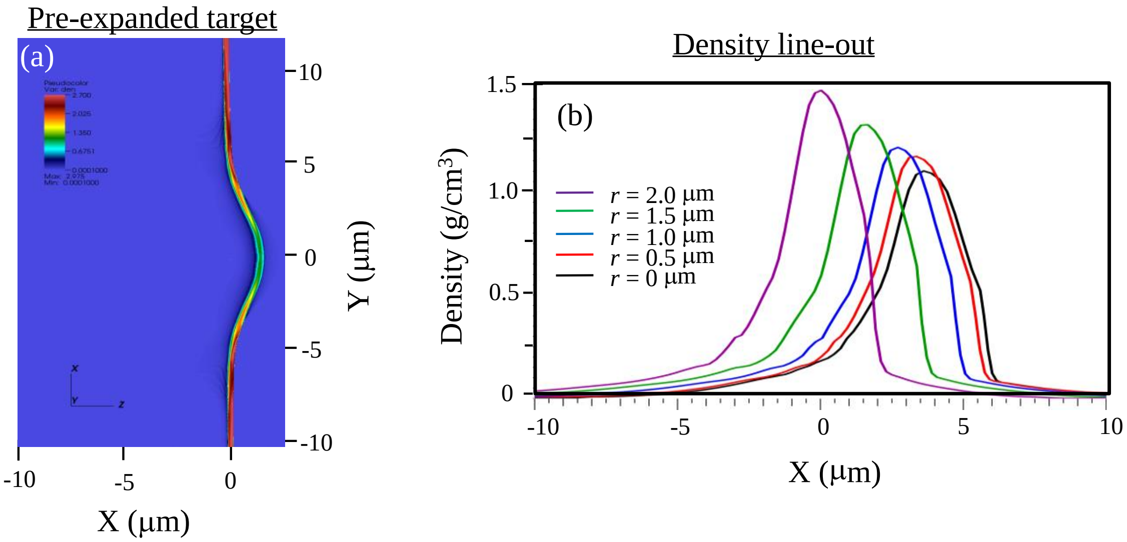

Supplementary Figure 7: 2D HYDRA code pre-expansion of 250 nm aluminum foil Supplementary figure 7a shows the 2D HYDRA rad-hydro code simulation result of 250nm thick aluminum foil pre-expansion due to Trident laser pedestal. Supplementary figure 7b shows the horizontal line-outs of pre-expanded plasma density profile at different radii. The plasma density profile near the middle of the laser focus peaks ~250n_{cr}, similar to the 1D results obtained from HELIOS hydro-code (see inset in Fig. 4f in the main article). 1D simulation uses average laser intensity whereas in 2D the local intensity on-axis is higher than 1D value. This effect compensates the transverse conduction in 2D (perhaps coincidently) resulting in comparable plasma pre-expansion both in 1D and 2D.

Electron charge density snapshot

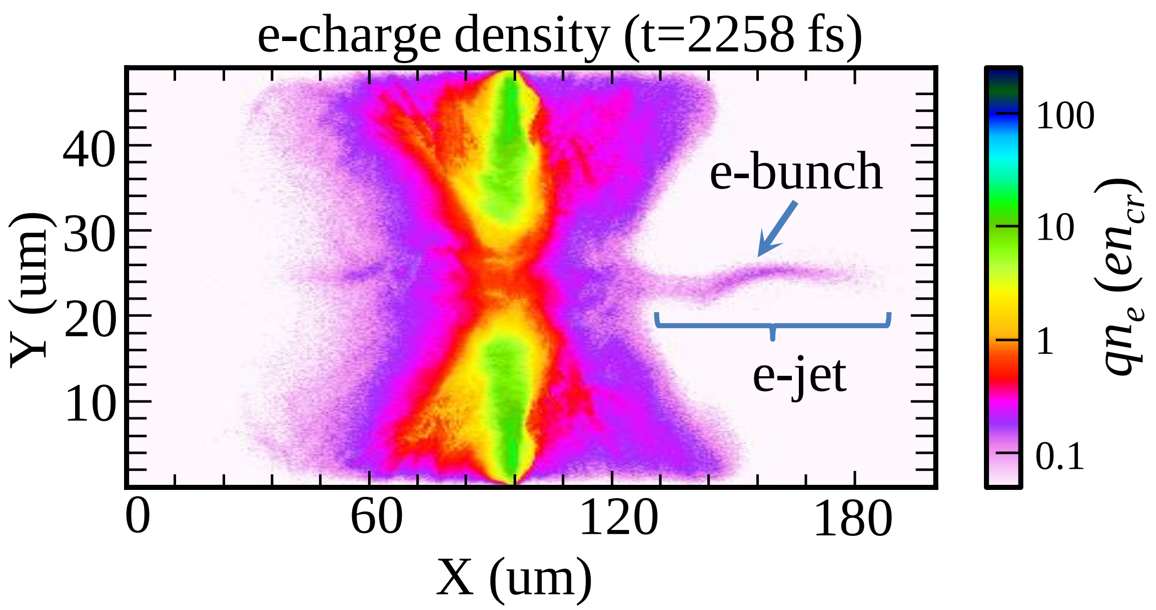

Supplementary Figure 8: Electron charge density snapshot Supplementary figure 8 shows the electron charge density snapshot at the end of the simulation. This distribution is similar to the ion distribution shown in the main manuscript. The electron jet and the slow-moving localized electron bunch are clearly seen in this figure.

Supplementary discussion

A concern arises as to whether the ion spectral peaking reported in the article could be due to multispecies light-sail RPA as explained in Supplementary Ref.[1] or leaky light-sail regime RPA in Supplementary Ref. [2]. In Supplementary Ref. [1], based on scaled simulations, the authors claim that their target remained highly reflective during the whole duration of the laser-plasma interaction. But in our case, both the experimental measurements and the full-scale simulations show that the plasma becomes relativistically transparent during the main laser pulse thus significantly different from Supplementary Ref. [1]. In leaky light-sail RPA (Supplementary Ref. [2]), the lightest species (protons) exhibits a spectral peak, not the bulk, heavier species (C). Also, the spectral peak is formed during the main laser pulse duration. In our work with Al & C foils (with proton contamination), there are spectral peaks in the bulk (and heaviest) species,

consistent with the simulation result indicating the influence of the late-time electron dynamics in self-generated fields on the bulk species. Hence it is not conceivable that either of these mechanisms play a major role in our case. The contribution of hole-boring radiation-pressure (HB-RPA) ion acceleration to the observed ion energy peak is minimal. For example, the maximum spectral red-shift of the reflected light in Fig.1h in the article is ~50 nm as the plasma moves away from the laser, which yields a maximum hole-boring velocity of $v_{hb}/c = d\lambda/2\lambda \cong 0.024$. Cold aluminum ions reflecting off this moving layer would gain a velocity of $2v_{hb}$ and energy of 29 MeV, which is 5 times lower than the measured 165 MeV ion peak.

Another concern is whether protons racing ahead of heavier ions could tamp and spectrally bunch the heavier ions. We have studied such issue in the past both experimentally and computationally in a controlled fashion using thin carbon nanofoils either with proton contamination or said contamination eliminated by preheating the target Supplementary Ref. [3]. The result was that the contaminant protons did indeed affect the carbon ion energy distribution, but it did so at the cutoff, that is energies in the 0.5-1 GeV (>42 MeV/nucleon equivalent to >1 GeV for Aluminum). Therefore, it does not seem reasonable that tamping of heavier ions by protons is dominant here, where the highest energy peaks are 310 MeV for 'Al' and 220 MeV for 'C'.

Another question is whether somehow different charge states of 'Al' (that is Al^{12+} and Al^{13+}) could race ahead of Al^{11+} and tamp the Al^{11+} energy distribution. In the case of C^{6+}, there is no higher C charge state to tamp the distribution; therefore at least in this case the concern is not pertinent. In the case of the f/3 laser focusing on Al (Experiment I - Table 1 in the article), we have simulated the problem with Al^{11+} (81.7%), Al^{12+} (18.1%) and Al^{13+} (0.2%) (Not shown here due to space constraints). The simulation showed no significant layering of the different Al charge states because their charge-to-mass ratios are very close to each other. It is therefore inconceivable that the Al^{11+} energy distribution is dominated by tamping from Al^{12+} and Al^{13+}.

We have also observed proton spectral peaks with a similar energy/nucleon as the heavier bulk species in the f/1.5 case (Experiment IV, V, and VII - Table 1 in the article) where it lies within the observable range of our TP detector. Previous measurements have shown that aluminum

targets could have protons in the bulk of the targets and not just at the surface Supplementary Ref. [4]. The spectrally peaked protons in our experiment may come from the bulk of the target and not from the surface due to TNSA. It is also conceivable that these proton spectral peaks could come from "buffered acceleration" involving relativistic transparency Supplementary Ref. [5]. But there are some remaining concerns to make such a claim. In Supplementary Ref. [5] only the lightest species (protons) showed a spectral peak while the heaviest species (C) energy spectrum was exponential. Moreover, the proton energy peak was at a much higher value than the average energy per nucleon of the bulk heavier species (C), different from our experimental results. To be conclusive on these hypotheses further computational and/or experimental studies are needed.

The late-time dynamics mediated by self-generated plasma fields explored here are complementary (not contradictory) to earlier work with BOA and ion acceleration in the relativistic transparency regime [6-8]. BOA refers to a brief period of enhanced ion acceleration upon the onset of relativistic transparency while the laser is on. Previous BOA simulations have not shown persistent ion spectral peaks. Here, we show reducing target pre-expansion and optimizing the laser-plasma interaction in the relativistic transparency regime mediated by self-generated plasma fields could lead to ion spectral bunching after the laser exits the plasma.

Supplementary References

1. Kar S*, et al.* Ion Acceleration in Multispecies Targets Driven by Intense Laser Radiation Pressure. *Physical Review Letters* **109**, 185006 (2012).

2. Qiao B*, et al.* Radiation-pressure acceleration of ion beams from nanofoil targets: the leaky light-sail regime. *Phys Rev Lett* **105**, 155002 (2010).

3. Jung D*, et al.* Laser-driven 1 GeV carbon ions from preheated diamond targets in the break-out afterburner regime. *Physics of Plasmas* **20**, 083103 (2013).

4. Hoffmeister G*, et al.* Influence of fs-laser desorption on target normal sheath accelerated ions. *Phys Rev Spec Top-Ac* **16**, (2013).

5. Dover NP*, et al.* Buffered spectrally-peaked proton beams in the relativistic-transparency regime. *arXiv:14063540v1*, (2014).

6. Yin L, Albright BJ, Bowers KJ, Jung D, Fernandez JC, Hegelich BM. Three-Dimensional Dynamics of Breakout Afterburner Ion Acceleration Using High-Contrast Short-Pulse Laser and Nanoscale Targets. *Physical Review Letters* **107**, 045003 (2011).

7. Jung D*, et al.* Efficient carbon ion beam generation from laser-driven volume acceleration. *New Journal of Physics* **15**, 023007 (2013).

8. Henig A*, et al.* Enhanced laser-driven ion acceleration in the relativistic transparency regime. *Phys Rev Lett* **103**, 045002 (2009).
